# Serum and cerebrospinal fluid neurofilament light chain and glial fibrillary acid protein levels in early and advanced stages of cerebral amyloid Angiopathy

**DOI:** 10.1186/s13195-024-01457-0

**Published:** 2024-04-23

**Authors:** Ingeborg Rasing, Sabine Voigt, Emma A. Koemans, Anna M. de Kort, Thijs W. van Harten, Ellis S. van Etten, Erik W. van Zwet, Erik Stoops, Cindy Francois, Bea H. Kuiperij, Catharina J.M. Klijn, Floris H.B.M. Schreuder, Louise van der Weerd, Matthias J.P. van Osch, Marianne A.A. van Walderveen, Marcel M. Verbeek, Gisela M. Terwindt, Marieke J.H. Wermer

**Affiliations:** 1 Department of Neurology, Leiden University Medical Center, Leiden, The Netherlands; 2 Department of Radiology, Leiden University Medical Center, Leiden, The Netherlands; 3 Department of Neurology, Donders Institute for Brain, Cognition and Behaviour, Radboud University Medical Center, Nijmegen, The Netherlands; 4 Department of Biomedical Data Sciences, Leiden University Medical Center, Leiden, The Netherlands; 5 ADx NeuroSciences, Ghent, Belgium; 6 Department of Human Genetics, Leiden University Medical Center, Leiden, The Netherlands; 7 Department of Laboratory Medicine, Radboud University Medical Center, Nijmegen, The Netherlands; 8 Department of Neurology, University Medical Center Groningen, Groningen, The Netherlands

**Keywords:** Cerebral amyloid angiopathy, Neurofilament light chain (NFL), Glial fibrillary acidic protein (GFAP), CAA CSVD score, MoCA

## Abstract

**Background:**

Neurofilament light chain (NFL) is a biomarker for neuroaxonal damage and glial fibrillary acidic protein (GFAP) for reactive astrocytosis. Both processes occur in cerebral amyloid angiopathy (CAA), but studies investigating the potential of NFL and GFAP as markers for CAA are lacking.

*We aimed to* investigate NFL and GFAP as biomarkers for neuroaxonal damage and astrocytosis in CAA.

**Methods:**

For this cross-sectional study serum and cerebrospinal fluid (CSF) samples were collected between 2010 and 2020 from controls, (pre)symptomatic Dutch-type hereditary (D-CAA) mutation-carriers and participants with sporadic CAA (sCAA) from two prospective CAA studies at two University hospitals in the Netherlands. NFL and GFAP levels were measured with Simoa-assays. The association between NFL and GFAP levels and age, cognitive performance (MoCA), CAA-related MRI markers (CAA-CSVD-burden) and Aβ40 and Aβ42 levels in CSF were assessed with linear regression adjusted for confounders. The control group was divided in age < 55 and ≥55 years to match the specific groups.

**Results:**

We included 187 participants: 28 presymptomatic D-CAA mutation-carriers (mean age 40 years), 29 symptomatic D-CAA participants (mean age 58 years), 59 sCAA participants (mean age 72 years), 33 controls < 55 years (mean age 42 years) and 38 controls ≥ 55 years (mean age 65 years).

In presymptomatic D-CAA, only GFAP in CSF (7.7*10^3^pg/mL vs. 4.4*10^3^pg/mL in controls; *P*<.001) was increased compared to controls. In symptomatic D-CAA, both serum (NFL:26.2pg/mL vs. 12.5pg/mL; *P*=0.008, GFAP:130.8pg/mL vs. 123.4pg/mL; *P*=0.027) and CSF (NFL:16.8*10^2^pg/mL vs. 7.8*10^2^pg/mL; *P*=0.01 and GFAP:11.4*10^3^pg/mL vs. 7.5*10^3^pg/mL; *P*<.001) levels were higher than in controls and serum levels (NFL:26.2pg/mL vs. 6.7pg/mL; *P*=0.05 and GFAP:130.8pg/mL vs. 66.0pg/mL; *P*=0.004) were higher than in pre-symptomatic D-CAA. In sCAA, only NFL levels were increased compared to controls in both serum (25.6pg/mL vs. 12.5pg/mL; *P*=0.005) and CSF (20.0*10^2^pg/mL vs 7.8*10^2^pg/mL; *P*=0.008). All levels correlated with age. Serum NFL correlated with MoCA (*P*=0.008) and CAA-CSVD score (*P*<.001). NFL and GFAP in CSF correlated with Aβ42 levels (*P*=0.01/0.02).

**Conclusions:**

GFAP level in CSF is an early biomarker for CAA and is increased years before symptom onset. NFL and GFAP levels in serum and CSF are biomarkers for advanced CAA.

**Supplementary Information:**

The online version contains supplementary material available at 10.1186/s13195-024-01457-0.

## Background

Cerebral amyloid angiopathy (CAA) is an important cause of lobar intracerebral hemorrhage (ICH) cognitive impairment in the elderly [1–4]. Sporadic amyloid β (Aβ)-type CAA pathology is caused by the accumulation of the Aβ protein in cortical and leptomeningeal arteries and arterioles [5, 6]. In the light of upcoming new therapeutic opportunities there is an urgent need for biomarkers that are able to detect (early) manifestations of CAA and are suitable for monitoring disease progression and treatment response. Important MRI-based markers of CAA pathology, such as lobar cerebral microbleeds and cortical superficial siderosis mainly represent advanced and irreversible CAA-related pathology [7]. Cerebrospinal fluid (CSF) and blood biomarkers are gaining increasing interest as new options to detect neuropathological processes, even in the presymptomatic disease stage. Dutch-type hereditary CAA (D-CAA) is a rare autosomal dominant form of CAA with an approximately twenty years earlier onset and a more aggressive disease course. This disease caused by a mutation in the Aβ precursor gene (APP), offers the unique opportunity to study the potential of new biomarkers from the presymptomatic phase up to advanced symptomatic stages of CAA [5].

CAA is closely associated with Alzheimer’s disease (AD). Approximately 50% of patients with AD have co-existing moderate-to-severe CAA pathology, although the cerebrovascular deposition of Aβ in CAA predominantly contains Aβ40 whereas in neuritic plaques in AD Aβ42 is the primary constituent [4, 8]. In AD, neurofilament light (NFL) levels and glial fibrillary acidic protein (GFAP) are promising serum and CSF biomarkers. These biomarkers are both non-disease specific, assumed to reflect neuroaxonal damage, reactive astrogliosis and neuroinflammation in several neurological disorders including AD [9, 10]. High plasma NFL levels are found in patient with symptomatic AD, compared with cognitively healthy controls [11]. Although NFL levels are known to increase with age, NFL serum levels are already increased in presymptomatic mutation-carriers with hereditary AD, almost a decade before estimated symptom onset [12, 13]. Until now, only two studies investigated NFL as biomarker in sCAA [14, 15]. One small exploratory study found increased CSF NFL levels in a group of 10 participants with sCAA compared to AD and control participants [14]. Another study that included 68 CAA-ICH cases from a Chinese prospective cohort, showed that increased serum NFL levels were associated with ICH recurrence compared to controls, independent of MRI SVD burden [15].

GFAP appears to be a sensitive biomarker for detecting and tracking astrogliosis even among individuals in the early stages of AD [16–18]. These findings suggest that astrocytic damage is already present in the presymptomatic phase of AD [10]. Moreover, a correlation between plasma GFAP levels and cortical Aβ deposition was reported in symptomatic AD [19]. However, these results should be interpreted considering the possible influence of co-existing large and small cerebral vessel disease and the age-related increase of GFAP expression by astrocytes [10, 20]. Whether GFAP levels are increased in CAA is unknown, but given the vascular phenotype of CAA with prominent astrogliosis, that is to be expected.

We aimed to investigate whether NFL and GFAP levels in serum and CSF, as biomarkers for neuroaxonal damage and astrocytosis, are abnormal in CAA and we assessed their correlation with age, cognitive function, MRI markers of CAA and Aβ levels in CSF.

## Methods

### Study population

We included (pre)symptomatic D-CAA mutation-carriers and participants with sCAA who participated between 2018 and 2020 in our ongoing prospective studies on disease progression and biomarkers in CAA (AURORA, FOCAS, BIONIC) and the completed CAVIA study (2010–2016). From these studies, we included all participants in whom a venous puncture and/or a lumbar puncture was performed.

Participants with D-CAA were recruited via the (outpatient) clinic of the Leiden University Medical Center (LUMC). Inclusion criteria were age ≥ 18 years and a DNA proven APP mutation, or a medical history of ≥ 1 lobar ICH(s) and ≥ 1 first-degree relative(s) with D-CAA. Symptomatic D-CAA was defined as a history of at least one symptomatic ICH (sICH). Participants with sCAA visited the (outpatient) clinic of the LUMC or the Radboud University Medical Center (RUMC) and were diagnosed with probable CAA by an experienced vascular neurologist and neuroradiologist based on the modified Boston criteria [21]. Control participants were visitors of the neurology outpatient clinic or were admitted to the RUMC in whom central nervous system (CNS) diseases were excluded after neurological examination and diagnostic workup. Cognitive impairment was an exclusion criterium. Controls were divided into < 55 years and ≥ 55 years of age to obtain matching age categories for the pre-symptomatic D-CAA carriers and participants with symptomatic D-CAA or sCAA. The cut-off point of 55 years was based on the mean age of index ICH in D-CAA and the age threshold in the modified Boston criteria for sCAA [21, 22].

We collected data on demographics, medical history and clinical symptoms for all D-CAA and sCAA participants by standardized questionnaires. Montreal Cognitive Assessment (MoCA) was used as a global cognitive screening test by trained staff at time of inclusion [23]. Demographic information was obtained for the control population. In this cohort, no information on cognitive function was available.

### Fluid biomarkers

Serum and CSF samples from the 3 cohorts were analyzed in the laboratories of ADx NeuroSciences, Ghent, Belgium. Serum and CSF NFL and GFAP levels were quantified using the commercially available single molecule array (Simoa)™ NF-Light Advantage Kit (Quanterix, catalogue nr. 103,186) and Simoa™ GFAP Discovery Kit (Quanterix, catalogue nr. 102,336) [24, 25]. A comprehensive description of the fluid biomarker analyses can be found in Supplementary Methods. Aβ1–40 and Aβ1–42 levels in CSF were quantified at the RUMC using Lumipulse® G fully automated immunoassays (Fujirebio, Ghent, Belgium).

### MRI assessment

The 3T MRI was performed in research setting on the same day as blood and CSF withdrawal was performed. The following MRI markers of CAA related brain injury were scored according to the Standards for Reporting Vascular changes on neuroimaging (STRIVE) criteria [26]: cerebral microbleeds (CMB), cortical superficial siderosis (cSS), white matter hyperintensities (WMH) and enlarged perivascular spaces in the centrum semi ovale (CSO-EPVS). Distribution of WMH was subdivided in periventricular WMH and deep WMH and scored according to the 4-point Fazekas rating scale [27]. PVS were rated using a validated visual rating scale (no PVS; ≤10 PVSs; 11–20 PVS; 21–40 PVS and > 40 PVS) [28]. The CAA related small vessel disease score (CAA CSVD score) was calculated for each participant. The CAA CSVD score consisted of lobar CMBs (2–4: 1 point, ≥ 5: 2 points), cSS (focal: 1 points, disseminated: 2 points), CSO-EPVSs (> 20: 1 point), and WMHs (deep WMH Fazekas score 2 or 3 and/or periventricular WMH Fazekas score 3: 1 point), with a higher score reflecting a more severe disease burden [29]. MR images were analyzed blinded for NFL and GFAP levels and clinical data. A single observer with over 5 years of experience in the field (EAK) scored all MRI markers and discussed her findings with a neuroradiologist with over 15 years of experience in the field (MAAvW) in case of uncertainty. Further details regarding the MRI protocol and assessment can be found in the Supplementary Methods.

### Statistical analysis

We investigated differences between the following groups: presymptomatic D-CAA versus controls < 55 years, symptomatic D-CAA versus controls ≥ 55 years, presymptomatic versus symptomatic D-CAA and sCAA versus controls ≥ 55 years. We performed multivariate linear regression analysis for NFL and GFAP levels for the pairwise comparison of groups with adjustment for age and sex. Second, we performed linear regression analysis to assess the association of the serum and CSF levels of NFL and GFAP with (1) age, (2) MoCA score, (3) CAA CSVD burden score, and (4) Aβ40 in CSF and (5) Aβ42 levels in CSF. We adjusted for age and sex in the analyses of the MoCA score, CAA CSVD burden score and the Aβ40 and Aβ42 levels. We assessed the correlation for NFL and GFAP levels in serum versus their levels in CSF by use of linear regression analysis.

## Results

We included 187 participants: 28 presymptomatic D-CAA mutation-carriers (mean age 40 years), 29 participants with symptomatic D-CAA (mean age 58 years), 59 participants with sCAA (mean age 72 years), 33 controls < 55 years (mean age 42 years) and 38 older controls ≥ 55 years (mean age 65 years), see Table 1 and Supplementary Fig. 1. In all participants with a history of symptomatic ICH (*n* = 59, 32%), the median time between ICH and blood and CSF withdrawal was 19.5 months from last ICH (range 2–87), see Supplementary Fig. 2. Serum samples were available for all sCAA participants, D-CAA mutation-carriers and controls. 51 CAA participants gave consent for a lumbar puncture (11 pre-symptomatic D-CAA (22%), 12 symptomatic D-CAA (24%) and 28 sCAA (55%)) and CSF was available for 53 controls. Age, sex and ICH presence were comparable for participants with (mean age 62 years, 51% female and 49% with ICH) and without lumbar puncture (mean age 60 years, 48% female and 52% with ICH).

**Table 1:** Baseline characteristics

	Presymptomatic D-CAA *n* = 28	Symptomatic D-CAA *n* = 29	Sporadic CAA *n* = 59	Controls < 55y *n* = 33	Controls ≥ 55y *n* = 38
Age, y, mean (range)	40.4 (27–55)	58.1 (43–74)	71.7 (57–86)	42.4 (27–54)	64.8 (55–85)
Women, n (%)	18 (64.3)	14 (48.3)	25 (42.4)	16 (48.5)	14 (36.8)
Education > 12y, n (%)	19 (67.9)	12 (41.4)	37 (62.7)^a^	-	-
Previous symptomatic ICH, n (%)	-	29 (100)	30 (50.8)	-	-
Time between ICH and blood/CSF withdrawal in months, median (range)	-	22 (2–85)	14 (2–87)	-	-
Cognitive testing performed, n (%)	28 (100)	29 (100)	51 (86.4)	-	-
MoCA, median (range)	28 (24-30)	27 (15-30)	25.5 (8-30)^b^	-	-
MRI data available, n (%)	25 (89.3)	27 (93.1)	55 (93.2)	-	-
Macrobleed count, median (range)	0 (0–0)	4 (1-26)	0 (0–13)	-	-
CAA CSVD score, median (range)	1 (0–4)	4 (3-60	4 (0–6)	-	-
CSF Aβ_40_ (pg/mL), median (range)^c^	2184 (832–3752)	1733.5 (910–2702)	6125 (1642–12,029)	7551.5 (2889–14,874)	9036 (3905–16,305)
CSF Aβ_42_ (pg/mL), median (range)^d^	102 (41–184)	76 (41–106)	312.5 (72–1088)	629 (233–1578)	681 (326–1265)
CSF p-tau (pg/mL), median (range)^e^	-	-	45.2 (24.5–207.1)	22.9 (11–49)	33.8 (18–94.7)

*Abbreviations: *Aβ = β-amyloid, *CAA *Cerebral amyloid angiopathy, *CSF *Cerebrospinal fluid, *CSVD *Cerebral small vessel disease, *D-CAA *Dutch cerebral amyloid angiopathy, *GFAP *Glial fibrillary acidic protein, *ICH *Intracerebral hemorrhage, *MoCA *Montreal Cognitive Assessment, *NFL *Neurofilament light, *p-tau *phosphorylate tau, *y *years

^a^ Missing data in 4 participants, ^b^ missing data in 8 participants

^c, d^ Presymptomatic participants with D-CAA *n* = 11, symptomatic participants with D-CAA *n* = 12, sCAA participants *n* = 28, controls < 55 *n* = 32, controls ≥ 55 *n* = 21

^e^ sCAA participants = 19, controls < 55 *n* = 31, controls ≥ 55 *n* = 21

### Serum and CSF NFL and GFAP levels in the early and advanced stages of CAA

**Fig. 1:**
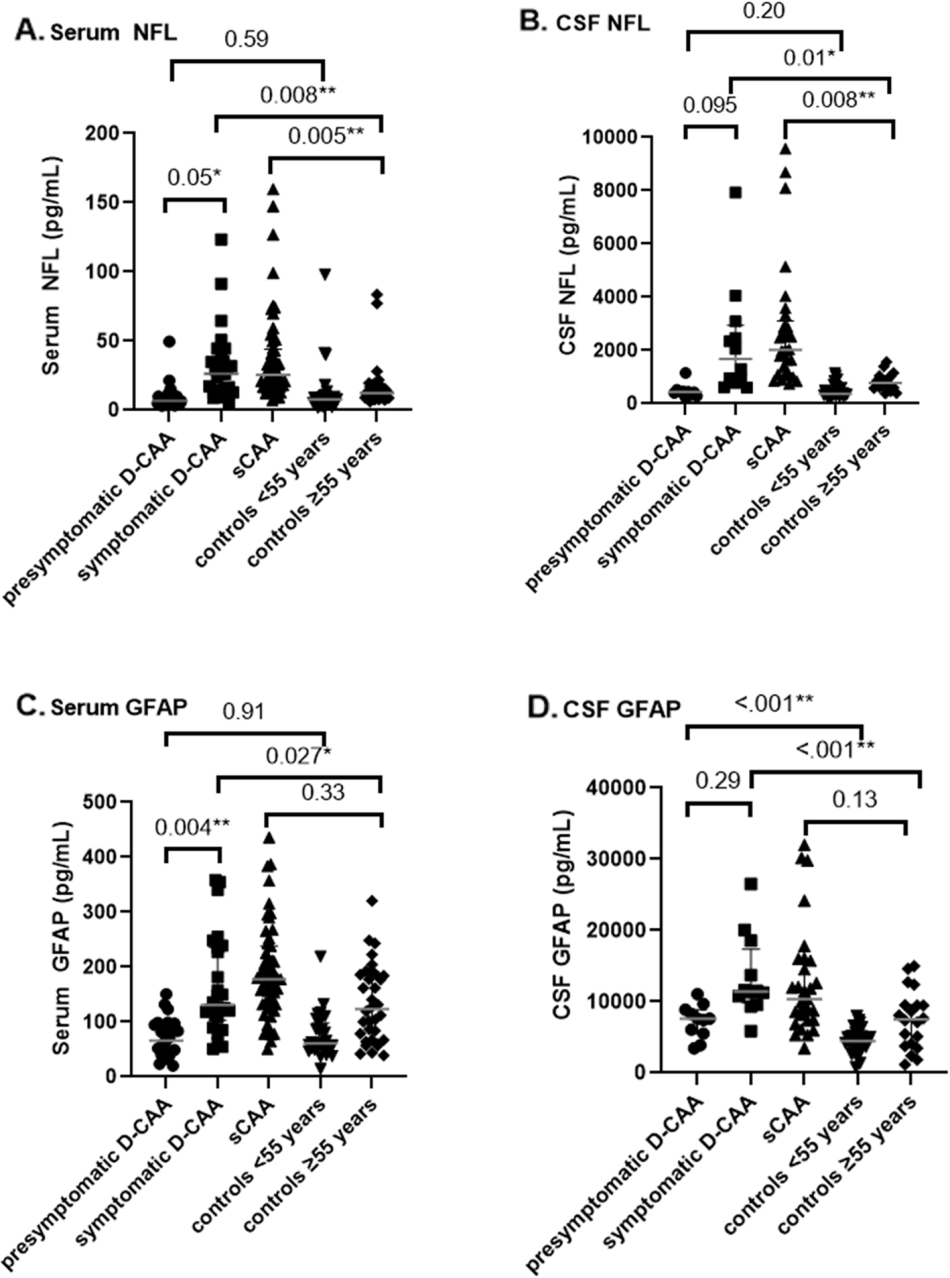
Shows the NFL an GFAP levels in D-CAA, sCAA and controls

NFL levels were similar in presymptomatic D-CAA and controls < 55 years in serum (6.7 pg/mL vs. 7.8 pg/mL; *P* = 0.59) and CSF (4.3*10^2^ pg/mL vs. 3.6*10^2^ pg/mL; *P* = 0.20) (Table 2; Fig. 1A and B). NFL levels were increased in symptomatic D-CAA vs. controls ≥ 55 years in serum (26.2 pg/mL vs. 12.5 pg/mL; *P* = 0.008) and CSF (16.8*10^2^ pg/mL vs. 7.8*10^2^ pg/mL; *P* = 0.01). NFL levels were higher in symptomatic versus presymptomatic D-CAA in serum (26.2 pg/mL vs. 6.7 pg/mL; *P* = 0.05) and CSF (16.8*10^2^ pg/mL vs. 4.3*10^2^ pg/mL; *P* = 0.095) and in participants with sCAA versus controls ≥ 55 years in both serum (25.6 pg/mL vs. 12.5 pg/mL; *P* = 0.005) and CSF (20.0*10^2^ pg/mL vs. 7.8*10^2^ pg/mL; *P* = 0.008). The GFAP levels were similar in presymptomatic D-CAA versus controls < 55 years in serum (66.0 pg/mL vs. 60.8 pg/mL; *P* = 0.91) but increased in CSF (7.7*10^3^ pg/mL vs. 4.4*10^3^ pg/mL; *P* = < 0.001, Fig. 1D). GFAP levels were increased in symptomatic D-CAA versus controls ≥ 55 years in serum (130.8 pg/mL vs. 123.4 pg/mL; *P* = 0.027) and CSF (11.4*10^3^ pg/mL vs. 7.5*10^3^ pg/mL; *P* < 0.001).

**Table 2:** Serum and CSF NFL and GFAP levels

	Presymptomatic D-CAA * n* = 28	Symptomatic D-CAA * n* = 29	Sporadic CAA * n* = 59	Controls <55 y * n* = 33	Controls ≥55 y * n* = 38
Serum NFL (pg/mL)^a^	6.72 (3.28 - 49.50)	26.20 (4.61 - 123.43)	25.56 (7.39 - 159.74)	7.78 (1.88 - 97.97)	12.46 (6.28 - 83.59)
CSF NFL*10^2^ (pg/mL)^b^	4.34 (2.30 - 11.54)	16.80 (6.06 - 79.23)	20.00 (7.36 - 95.79)	3.63 (2.14 - 11.47)	7.76 (3.76 - 15.72)
Serum GFAP (pg/mL)^c^	66.03 (20.16 - 150.46)	130.75 (51.21 - 358.70)	177.89 (51.18 - 436.01)	60.75 (14.45 - 218.84)	123.37 (38.82 - 320.52)
CSF GFAP*10^3^ (pg/mL)^d^	7.69 (3.46 - 11.03)	11.41 (5.87 - 26.50)	10.93 (3.48 - 32.01)	4.44 (0.77 - 7.98)	7.51 (1.19 - 14.99)

^a^Presymptomatic participants with D-CAA *n*=28, symptomatic participants with D-CAA *n*=28, sCAA participants *n*=56, controls <55 *n*=29, controls ≥55 *n*=37

^b^Presymptomatic participants with D-CAA *n*=10, symptomatic participants with D-CAA *n*=12, sCAA participants *n*=28, controls <55 *n*=31, controls ≥55 *n*=18

^c^Presymptomatic participants with D-CAA *n*=25, symptomatic participants with D-CAA *n*=26, sCAA participants *n*=51, controls <55 *n*=31, controls ≥55 *n*=34

^d^Presymptomatic participants with D-CAA *n*=11, symptomatic participants with D-CAA *n*=12, sCAA participants *n*=28, controls <55 *n*=32, controls ≥55 *n*=20

*Abbreviations:* *CAA *cerebral amyloid angiopathy, *CSF *cerebrospinal fluid, *NFL *neurofilament light chain, *D-CAA *Dutch Cerebral Amyloid Angiopathy, *GFAP *glial fibrillary acidic protein, *y *years

GFAP levels in serum were higher in symptomatic versus presymptomatic D-CAA (130.8 pg/mL vs. 66.0 pg/mL; *P* = 0.004) but not in CSF (11.4*10^3^ pg/mL vs. 7.7*10^3^ pg/mL; *P* = 0.29). GFAP levels were similar in sCAA versus controls ≥ 55 years in serum (177.9 pg/nL vs. 123.4 pg/nL; *P* = 0.33) and CSF (10.9*10^3^ pg/mL vs. 7.5 pg/mL; *P* = 0.13, Fig. 1C and D).

### Association of serum and CSF NFL and GFAP levels with age, cognition, CAA burden on MRI and CSF amyloid-βlevels

**Fig. 2:**
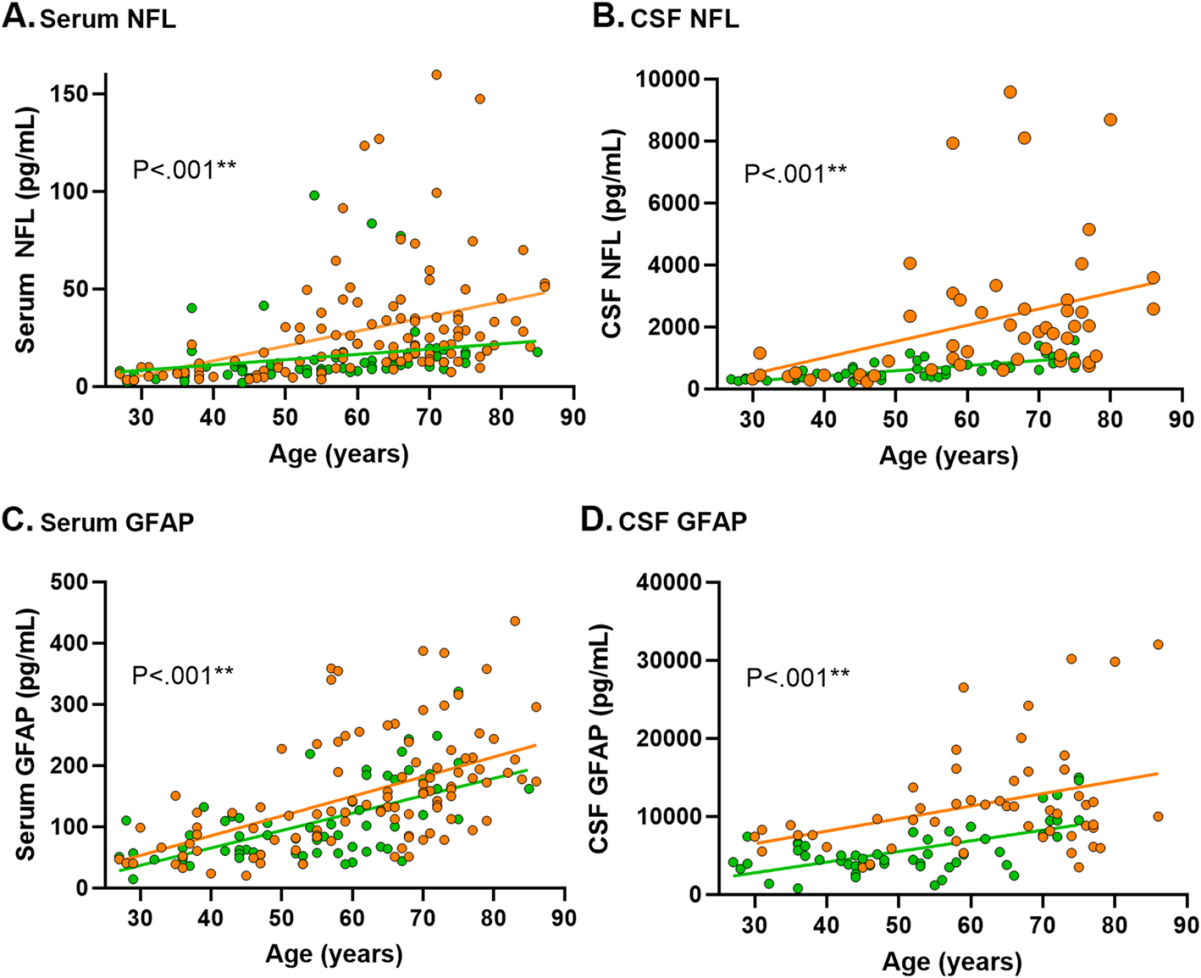
Shows the association of the biomarker levels with age. *P*-values are based on the linear regression analysis of all participants

Increasing NFL levels in serum (β [95%CI] = 0.60 [0.37–0.83]; *P* < 0.001), NFL levels in CSF (β [95%CI] = 47.38 [26.91–67.85]; *P* < 0.001), GFAP levels in serum (β [95%CI] = 3.30 [2.58–4.02] *P* < 0.001) and GFAP levels in CSF (β [95%CI] = 207.07 [140.02–274.12]; *P* < 0.001) were all correlated with increasing age (Fig. 2).

**Fig. 3:**
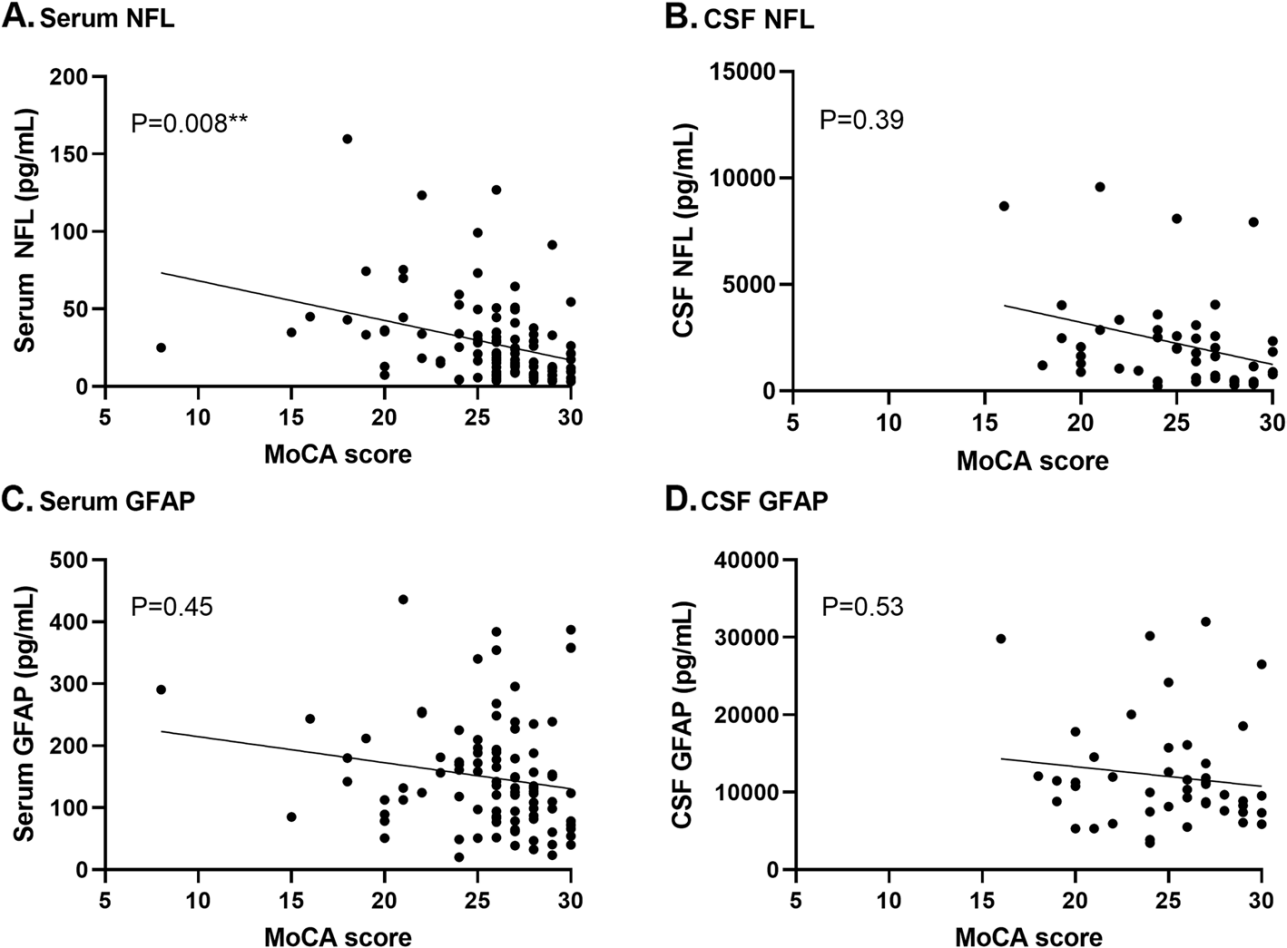
Shows *n*=108 participants in whom the MoCA was performed (28 presymptomatic D-CAA, 29 symptomatic D-CAA and 51 sporadic CAA)

Increasing levels of NFL in serum were associated with decreasing MoCA scores (β [95%CI] = -1.97 [-3.42 – -0.52]; *P* = 0.008) whereas GFAP levels in serum and NFL and GFAP levels in CSF were not (Fig. 3A-D, Supplementary Fig. 4).

**Fig. 4:**
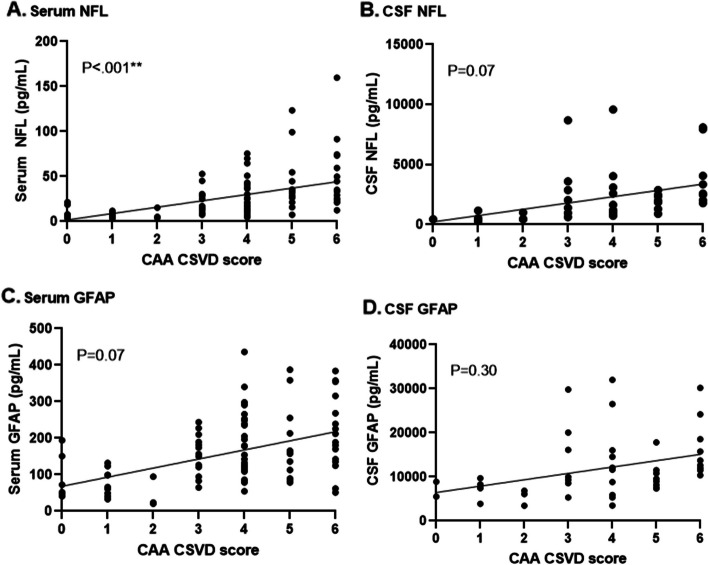
Shows *n*=107 participants in whom a 3 Tesla MRI was performed (*n*=25 presymptomatic D-CAA, *n*=27 symptomatic D-CAA and *n*=55 sporadic CAA)

Increasing NFL levels in serum (β [95%CI] = 6.03 [2.72–9.35]; *P* = < 0.001) correlated with higher CAA CSVD scores, whereas GFAP levels in serum (β [95%CI] = 11.10 [-0.90–23.10]; *P* = 0.07), NFL levels in CSF (β [95%CI] = 429.55 [-38.35–897.45]; *P* = 0.07) and GFAP levels in CSF (β [95%CI] = 775.65 [-721.59–2272.90]; *P* = 0.30) did not show a clear association with the CSVD score, Fig. 4A-D, Supplementary Fig. 5).

**Fig. 5:**
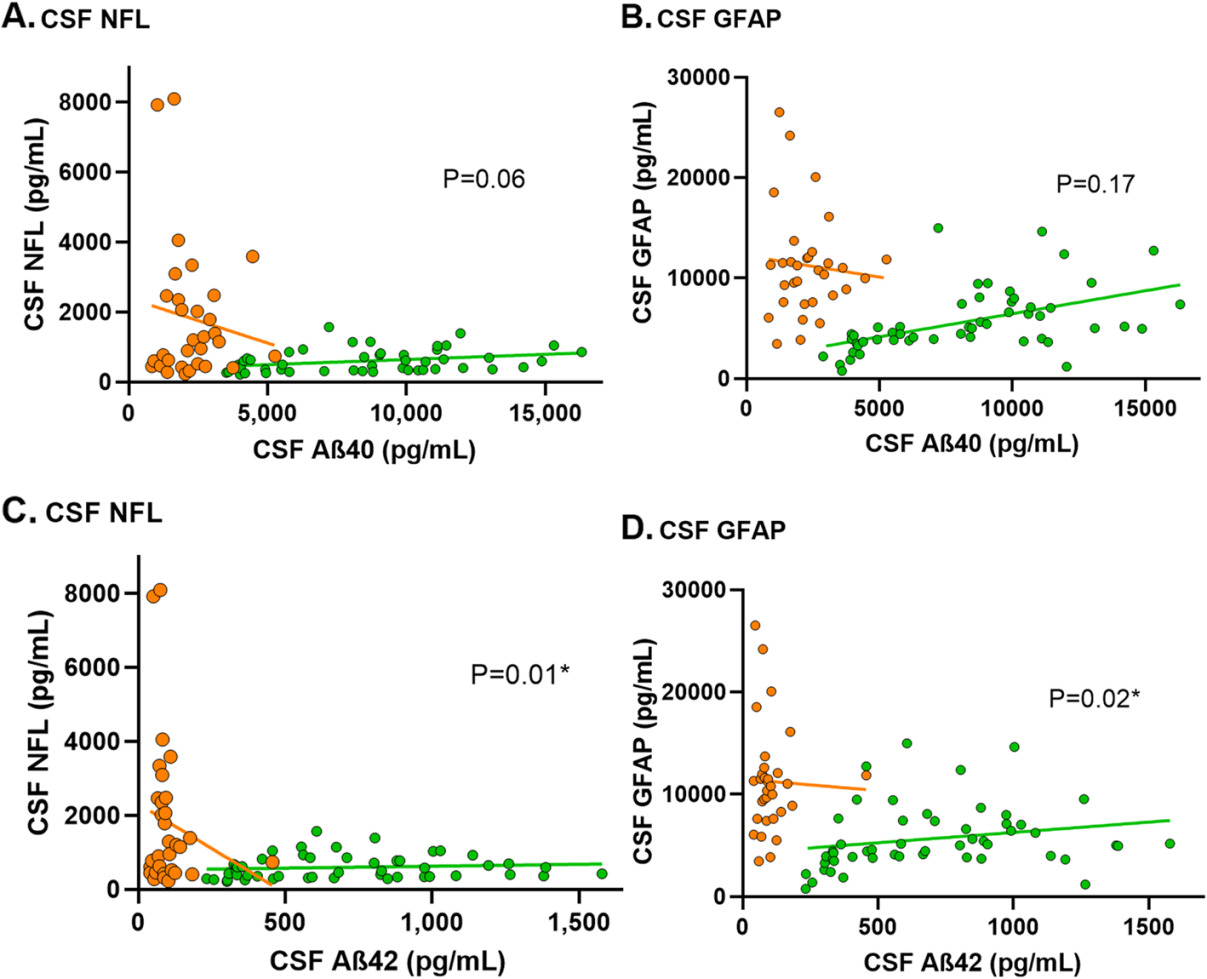
Shows *n*=104 participants (11 presymptomatic D-CAA, 12 symptomatic D-CAA, 28 sporadic CAA, 32 controls <55 years and 21 controls ≥55 years). *P*-values are based on the linear regression analysis of all participants

Aβ40 and Aβ42 levels in CSF were decreased in presymptomatic D-CAA, symptomatic D-CAA and sCAA in comparison to controls (Table 1). Increasing levels of NFL and GFAP in CSF were correlated with decreasing Aβ40 and Aβ42 levels in CSF although the correlation was only statically significant for the correlation with Aβ42 (NFL: β [95%CI] = -1.08 [-1.91 – -0.24]; *P* = 0.01 and GFAP: β [95%CI] = -3.16 [-5.89 – -0.43]; *P* = 0.02, Fig. 5).

## Discussion

In this large prospective cohort of both hereditary and sCAA participants we found that (1) GFAP in CSF is increased in the early presymptomatic stage of CAA, (2) NFL and GFAP levels in serum and CSF are increased in either sporadic or more severe hereditary symptomatic stages of CAA and (3) increasing levels of NFL and GFAP are associated with increasing age, decreasing MoCA score, increasing CAA SVD score on MRI and decreasing Aβ levels in CSF. Although both markers are not-CAA specific, these results suggest that GFAP in CSF is a biomarker for early CAA pathology and both NFL and GFAP in serum and CSF are markers for advanced stages of CAA.

Our findings that NFL is increased in participants with symptomatic D-CAA as well as sCAA compared to controls confirm the results of a small previous exploratory cross-sectional study that found increased NFL levels in CSF in 10 participants with sCAA compared to participants with AD and controls [14]. Our results are also consistent with previous studies that investigated NFL as a promising biomarker in other neurodegenerative and neurovascular diseases. CAA is closely related to AD. In both diseases Aβ deposition plays a crucial role although the subsequent mechanisms of brain injury are different [8]. In AD, NFL levels in plasma and CSF were shown to be higher than in healthy controls and CSF NFL was associated with cognitive decline, white matter changes and brain atrophy [11, 30]. Also, in non-demented elderly, NFL levels in serum were associated with SVD markers on MRI and impaired processing speed [31, 32]. Moreover, in CADASIL, the most prevalent form of hereditary SVD, serum NFL levels have been correlated with disease severity (microbleed and lacune count), disease progression and survival [33]. In line with these findings, we found a correlation between NFL levels in serum with the CAA CSVD score. For MoCA, the correlation was present, but less pronounced. There is growing evidence that cognition in CAA appears to be more specifically affected in the domains of executive functioning and processing speed [3, 34, 35].The rather unspecific nature of the MoCA score might explain why the correlation with serum NFL was not very robust. This explanation might also apply to GFAP in serum and CSF NFL and GFAP.

GFAP levels were increased in CSF but not in serum of presymptomatic mutation-carriers with D-CAA. This difference could be explained by the proximity of CSF to cerebral neuropathology with subsequent diluted effects in serum. Furthermore, GFAP levels were increased in symptomatic D-CAA compared to controls in both serum and CSF. This is in line with a previous study in four transgenic mouse models of amyloid deposition, that found that CAA pathology causes loss of GFAP-positive cells [36]. However, in sCAA, GFAP levels were somewhat higher but not statistically significant increased compared to controls. We do not fully understand this finding. Possible explanations might be the limited sample size or residual confounding of aging effects.

Recent studies indicated that GFAP is promising biomarker for several neurological diseases other than CAA [37]. A previous study reported that GFAP levels were increased in serum and CSF of patients with AD [16]. Based on our results, it might be possible that at least a part of the increase of NFL or GFAP in AD is due to co-existing CAA pathology, since this is frequently observed in brains of patients with AD [38, 39]. To our knowledge there have been no previous studies investigating serum and CSF GFAP levels in CAA.

In contrast with GFAP levels in CSF, NFL levels were not increased in the presymptomatic phase of D-CAA. Because NFL is mainly a biomarker for neurodegeneration, this might indicate that neurodegeneration occurs at later stages of the CAA disease cascade compared to neuroinflammation and perivascular astrocyte activation.

Strengths of our study are our unique hereditary CAA population including presymptomatic carriers, which makes it possible to investigate the early asymptomatic stages of disease in persons with a definite diagnosis of CAA. Second, the participants with D-CAA are relatively young with limited coexisting age-related pathology. Third, all data of participants with sCAA and D-CAA mutation-carriers were prospectively collected using a standardized study protocol with all study components performed at the same study visit. Moreover, we used state-of-the-art ultra-sensitive Simoa to reliably asses NFL and GFAP levels in both CSF and peripheral blood [40].

Our study has limitations. First, because not all participants consented for lumbar puncture, the number of included participants with CSF was relatively small. This might explain why we did not find significant associations between NFL and GFAP CSF levels, and cognitive performance and the CAA CSVD score. Second, we did not have data on MRI markers or cognitive performance for the control group. Third, the sample sizes of our CAA groups were not sufficient to allow subgroup analyses with tauopathy positive (increased phosphorylated tau (p-tau)) and tauopathy negative participants to assess the possible influence of co-existing AD. Fourth, the control participants in our study were not true healthy controls since they visited the outpatient neurology clinic with symptoms. However, no CNS diseases were diagnosed in these persons after careful evaluation. Fifth, blood and CSF samples of participants with sCAA were collected in two different centers. We tried to minimize differences between the centers by uniform collection, storage and grouped analyses and use of the same pre-analytical protocol. Sixth, we did not correct for multiple comparisons as this was an explorative study. Finally, as a consequence of the cross-sectional study design, we were not able to assess the association of NFL and GFAP with disease progression. Our results, however, do suggest an association with disease severity on a group level with higher NFL and GFAP levels in participants with symptomatic versus presymptomatic D-CAA.

## Conclusions

Our study shows that GFAP in CSF is an early indicator of CAA related pathology and is increased years before ICH occurs. NFL and GFAP levels in serum and CSF are biomarkers reflecting neurodegeneration and reactive astrocytosis in advanced CAA. Both NFL and GFAP correlate with age, cognition, CAA related changes on MRI and Aβ in CSF. Future longitudinal studies are needed to investigate the prognostic value of NFL and GFAP and their potential to monitor therapeutic treatment responses in CAA pathology.

### Supplementary Information

**Supplementary Material 1.**

## Data Availability

No datasets were generated or analysed during the current study.

## Abbreviations

CSF: Cerebrospinal fluid
CAA: Cerebral amyloid angiopathy
D-CAA: Dutch-type hereditary
sCAA: Sporadic CAA
NFL: Neurofilament light chain
GFAP: Glial fibrillary acidic protein
MoCA: Montreal cognitive assessment
CSVD: Cerebral small vessel disease
Aβ: Amyloid-β
APP: Amyloid precursor protein
AD: Alzheimer’s disease
sICH: Symptomatic intracerebral hemorrage
CNS: Central nervous system
Simoa: Single molecule array
CMB: Cerebral microbleeds
cSS: Cortical superficial siderosis
WMH: White matter hyperintensities
CSO: Centrum semi ovale
EPVS: Enlarged perivascular spaces
