# Correction: Serum and cerebrospinal fluid neurofilament light chain and glial fibrillary acid protein levels in early and advanced stages of cerebral amyloid angiopathy

**DOI:** 10.1186/s13195-024-01518-4

**Published:** 2024-07-06

**Authors:** Ingeborg Rasing, Sabine Voigt, Emma A. Koemans, Anna M. de Kort, Thijs W. van Harten, Ellis S. van Etten, Erik W. van Zwet, Erik Stoops, Cindy Francois, H. Bea Kuiperij, Catharina J.M. Klijn, Floris H.B.M. Schreuder, Louise van der Weerd, Matthias J.P. van Osch, Marianne A.A. van Walderveen, Marcel M. Verbeek, Gisela M. Terwindt, Marieke J.H. Wermer

**Affiliations:** 1 Department of Neurology, Leiden University Medical Center, Leiden, The Netherlands; 2 Department of Radiology, Leiden University Medical Center, Leiden, The Netherlands; 3 Department of Neurology, Donders Institute for Brain, Cognition and Behaviour, Radboud University Medical Center, Nijmegen, The Netherlands; 4 Department of Biomedical Data Sciences, Leiden University Medical Center, Leiden, The Netherlands; 5 ADx NeuroSciences, Ghent, Belgium; 6 Department of Human Genetics, Leiden University Medical Center, Leiden, The Netherlands; 7 Department of Laboratory Medicine, Radboud University Medical Center, Nijmegen, The Netherlands; 8 Department of Neurology, University Medical Center Groningen, Groningen, The Netherlands

**Correction:** ***Alz Res Therapy*** **16** **, 86 (2024)**

10.1186/s13195-024-01457-0

Following publication of the original article [1], important inaccuracies were observed:

1. In the funding paragraph, the following important funding statement was intended to be mentioned:

‘Sabine Voigt is supported by the Netherlands Heart Foundation (2016T86) and the Netherlands Organization for Health Research and Development (ZonMw/Hersenstichting) [project number DR-2019-00299].

2. The tenth co-author’s name is H. Bea Kuiperij, not Bea H. Kuiperij.

The original article [1] has been corrected.
