# Supplementary material for: Serum and cerebrospinal fluid neurofilament light chain and glial fibrillary acid protein levels in early and advanced stages of cerebral amyloid Angiopathy
Source: Alzheimers Res Ther. 2024 Apr 23;16:86. doi: 10.1186/s13195-024-01457-0 (PMC11036675; doi:10.1186/s13195-024-01457-0)
Supplement: Supplementary file 1 — Supplementary Material 1. [file 13195_2024_1457_MOESM1_ESM.pdf]

SUPPLEMENTARY MATERIAL

Supplementary Figure 1. Flowchart of participants

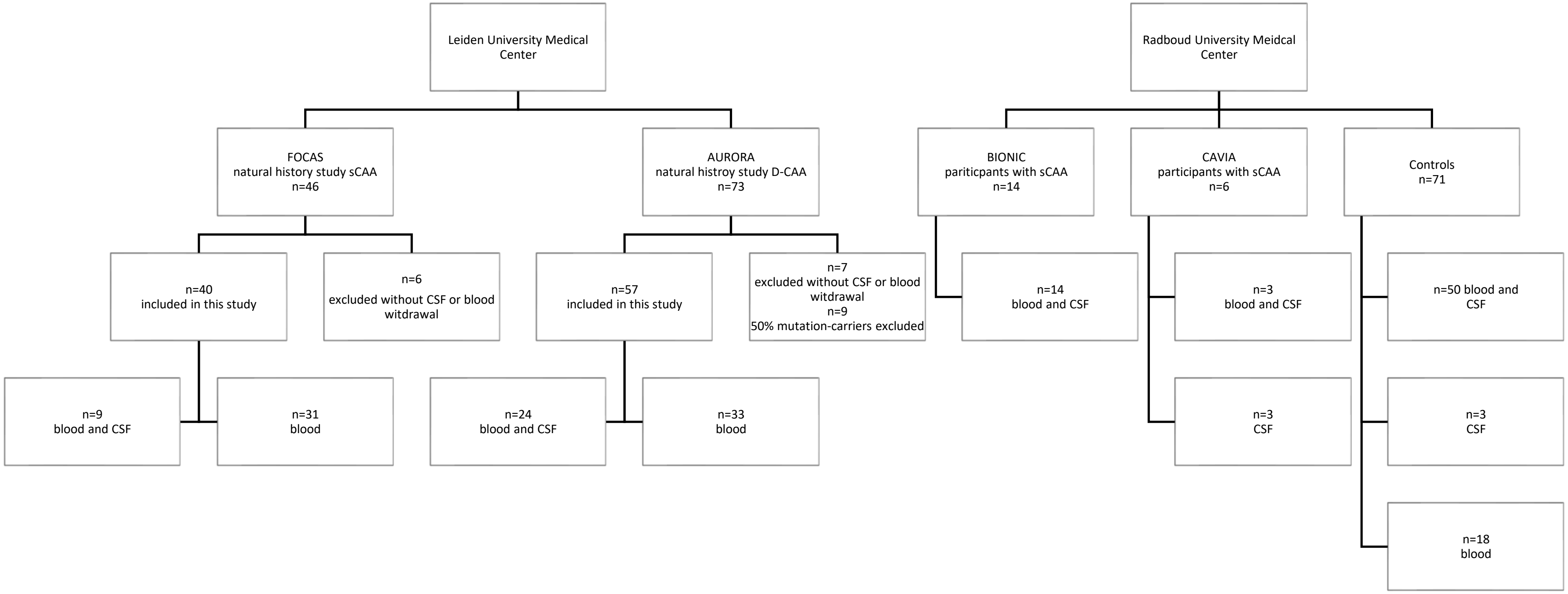

Supplementary Figure 2. Correlation between biomarkers and time since ICH

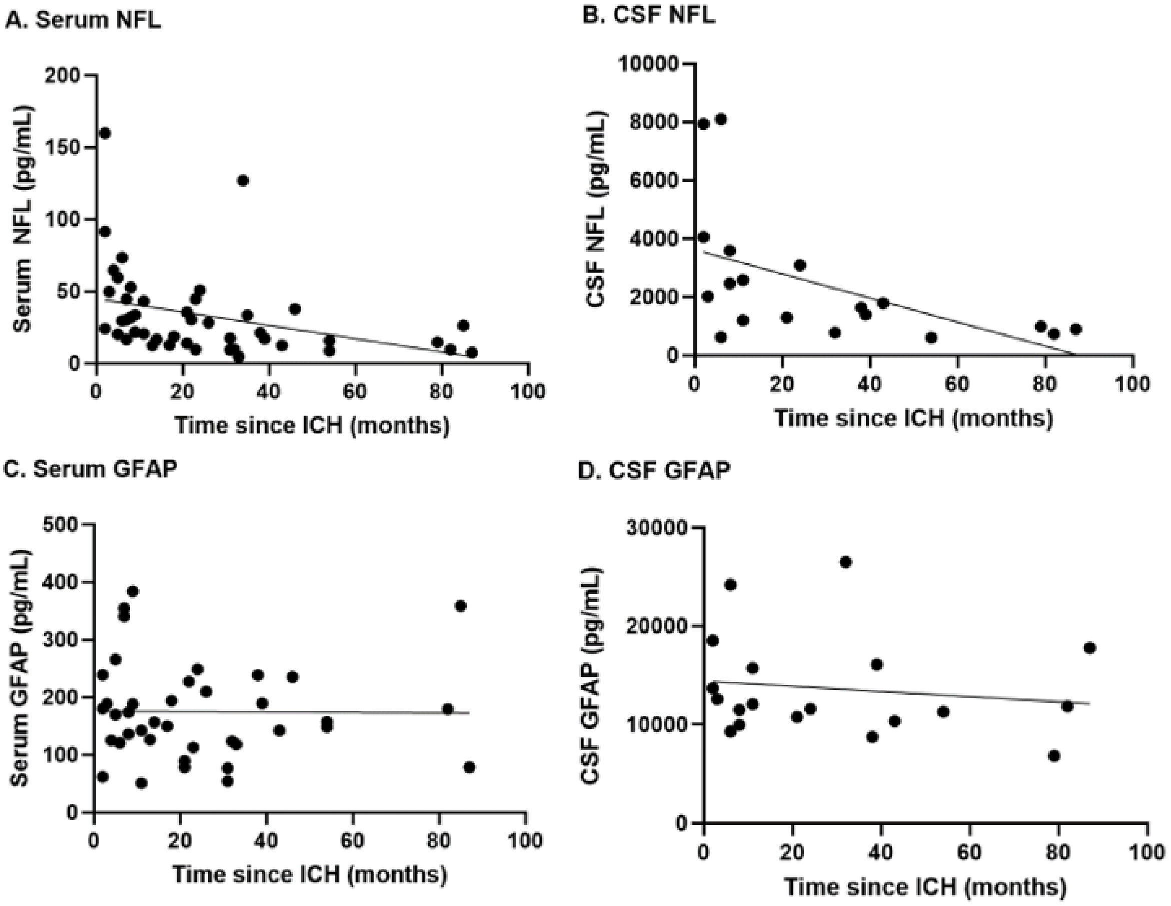

Supplementary Figure 3. Correlation between CSF and serum measurements

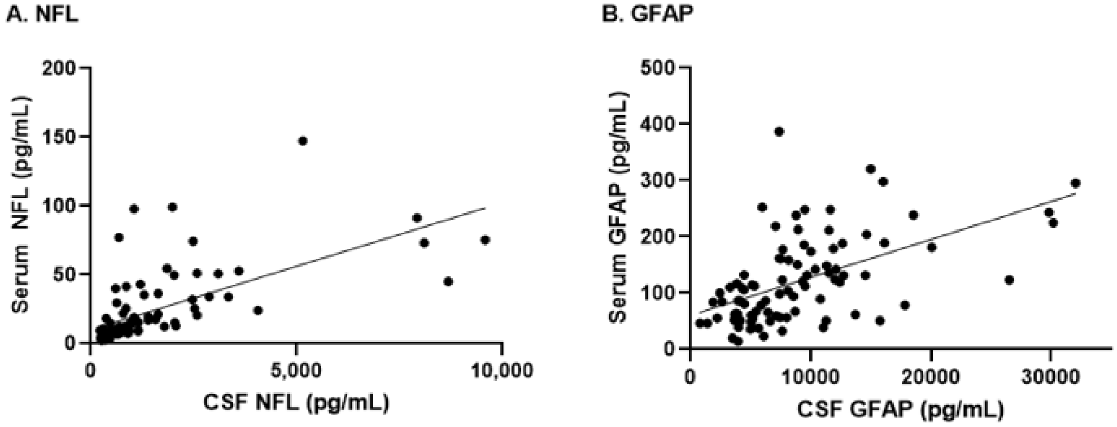

Supplementary Figure 4. Associations of NFL and GFAP levels with the MoCA score

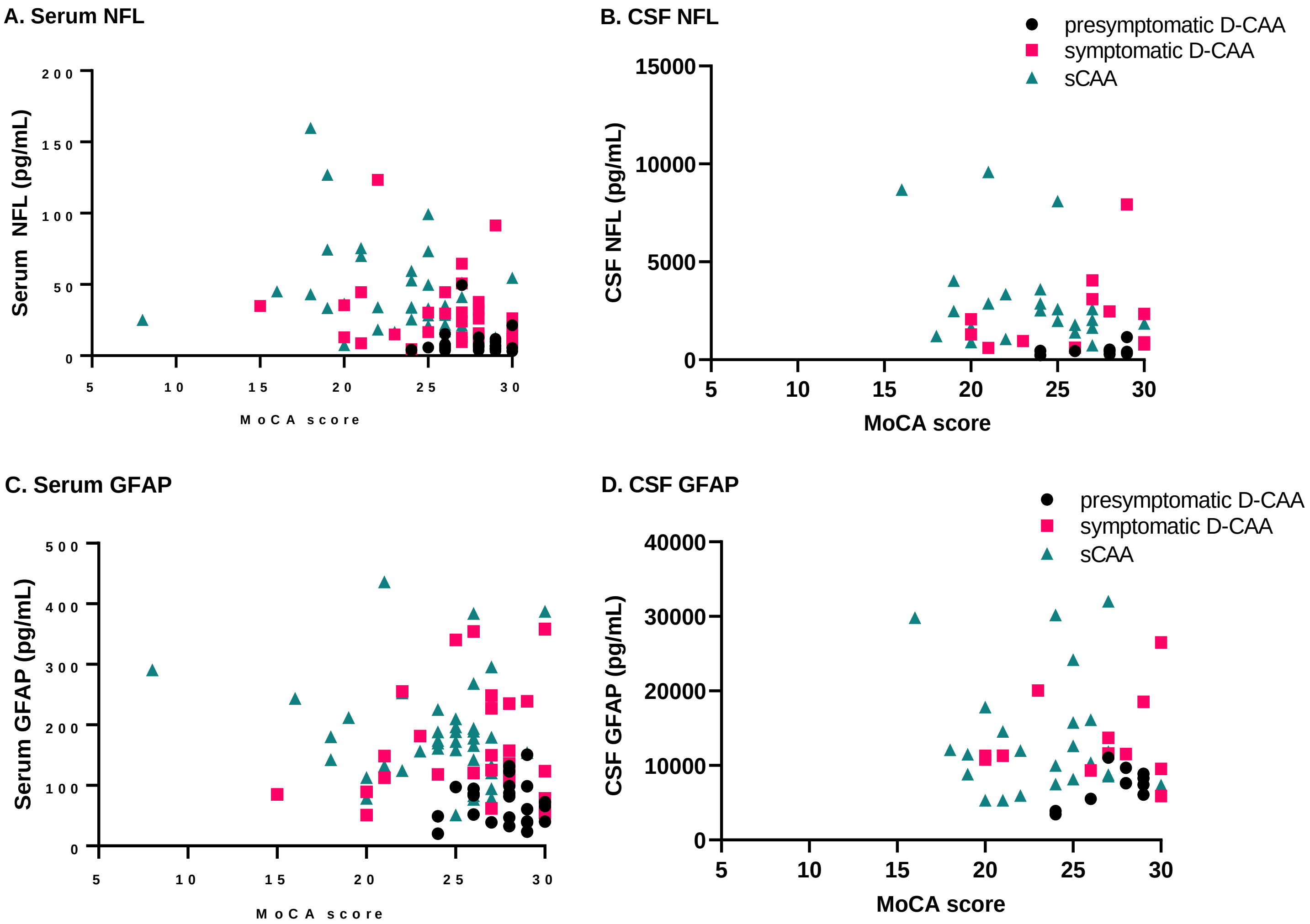

Supplementary Figure 5. Associations of NFL and GFAP levels with the CAA CSVD score

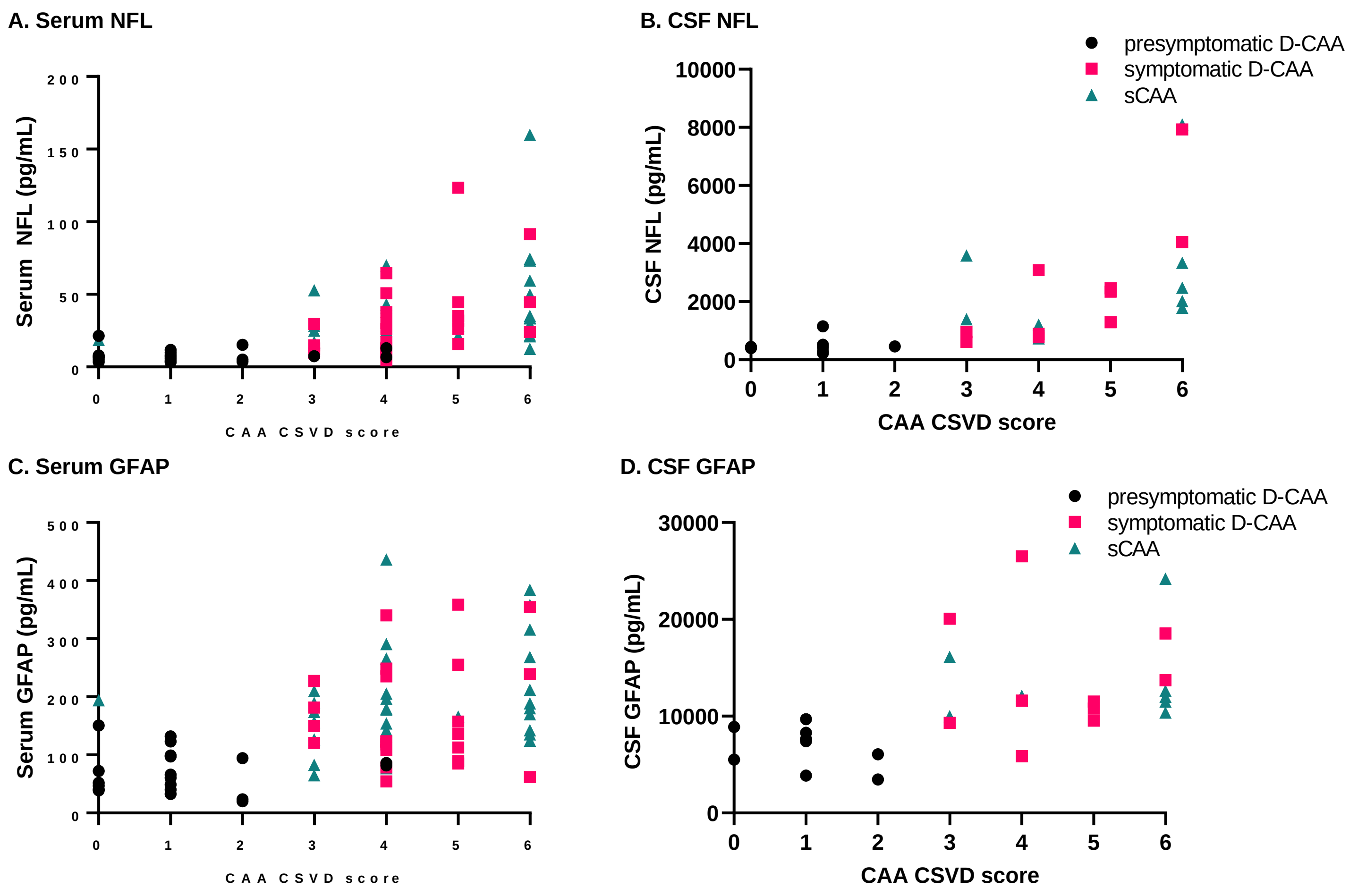

Supplementary Methods

Serum NFL and GFAP measurements

At baseline, blood was withdrawn via standard vena puncture and stored at room temperature for a maximum of 2 hours until centrifuging (2350 relative centrifugal force (RCF) for 10 minutes at 20° C) and storage in aliquots in polypropylene tubes at -80°C. The commercially available Simoa™ NF-Light Advantage Kit (Quanterix) and Simoa™ GFAP Discovery Kit (Quanterix) were used according to manufacturer's instructions except for GFAP serum analysis where a 8 fold sample dilution was applied due to sample volume restrictions, as previously described.[1] The serum samples were randomized and centrifuged prior to use (3 min; 14000g) followed by manual 4 fold respectively 8 fold dilution for NFL and GFAP into a 96 well microplate. Next, sample testing was done in duplicate over two consecutive test runs. For the GFAP analysis the samples received an additional freeze-thaw step compared to the NFL analysis. Samples with %CV>20 were excluded from the analysis. An average intra-assay %CV of 3.4% (range 0.0- 17.8) for NFL and 5.8% (range 0.0 - 19.8) for GFAP were obtained, samples with an intra-assay CV >15% were re-run for NFL and then excluded from further analyses when intra-assay was >20%.

CSF NFL and GFAP measurements

We collected CSF samples of all participants who consented to undergo a lumbar puncture, all samples were obtained under standardized conditions at the same time frame. CSF was collected in polypropylene tubes and transferred to the laboratories within 30 minutes. We centrifuged (2350 RCF for 10 minutes at 20°C) the samples and stored them in aliquots in polypropylene tubes at -80°C. CSF NFL and GFAP levels were quantified using Simoa Kits, as mentioned above according to the manufacturer's instructions. The CSF samples were randomized and centrifuged prior to use (3 min; 14000g) followed by manual 100 fold respectively 40 fold dilution for NFL and GFAP into a 96 well microplate. Next, sample testing was done in duplicate in a single test runs.

Intra-assay CVs were 2.8% (range 0.0 - 14.4) for NFL and 2.9% (range 0.1 - 14.6) for GFAP, samples with an intra-CV >20% were excluded after a re-run for all samples with an intra-assay CV >15%. Aβ40 and Aβ42 levels in CSF were quantified using Lumipulse® G fully automated immunoassays (Fujirebio, Gent, Belgium).

MRI protocol

Leiden:

MRI was performed on a 3.0 Tesla MRI scanner (Philips Achieva, Best, the Netherlands) in all D-CAA and sCAA participants and data was acquired using a standard 32-channel head coil. Three-dimensional T1 weighted images were acquired with the following parameters: repetition time(TR)/ echo time (TE) = 9.7/4.6ms, flip angle 7 degrees, 130 slices with no interslice gap and a field of view (FOV) of 217 x 172 x 156 mm with a voxel size of 1.2 x 1.2 x 1.2 mm, resulting in a scan duration of 2:48 min. T2 weighted images were acquired with the following parameters: TR/ TE = 4744/80ms, flip angle 90 degrees, 48 slices with no interslice gap and an FOV of 220 x 176 x 144 mm with a voxel size of 0.5 x 0.6 x 3 mm, resulting in a scan duration of 2:13 min. Three dimensional Fluid Attenuated Inversion Recovery (FLAIR) images were acquired with the following parameters: TR/TE = 4800/280ms, inversion time (TI) 1650ms , 321 slices with no interslice gap and an FOV of 250 x 250 x 180 mm with a voxel size of 1.1 x 1.1 x 0.6 mm, resulting in a scan duration of 4:43 min. Susceptibility weighted images (SWI) were acquired using the following parameters: TR/TE = 31/7.2ms, flip angle 17 degrees, 130 slices and an FOV of 230 x 190 x 130 mm with a voxel size of 0.6 x 0.6 x 1 mm resulting in a scan duration of 3:31 min.

Nijmegen:

The CAA participants from the CAVIA study underwent a 1.5 or 3.0 Tesla MRI of the brain using different MRI systems with varying protocols in the RUMC or referral hospitals, which at least included T2*-weighted images or susceptibility-weighted imaging sequence (SWI) images, fluid-attenuated inversion recovery (FLAIR) and T2 sequences. The BIONIC participants underwent a 3.0

Tesla MRI scan (Siemens Magneto, Siemens Healthcare,Erlangen, Germany) with a 32-channel head coil. Participants were examined using a comprehensive protocol, and the for the current study, the 3D multi-echo gradient echo T2*-weighted sequence (voxel size 0.8 x 0.8 x0.8 mm), the 3D T2-weighted sequence (voxel size 0.8 x 0.8 x 0.8 mm) and 3D fluid-attenuated inversion recovery (FLAIR) sequence (voxel size 0.8 x 0.8 x0.8 mm) were analyzed. Magnitude and phase data from the multi-echo gradient sequence was processed to a SWI using the "Contrast-weighted, Laplace-unwrapped, bipolar multi-Echo, ASPIRE-combined, homogeneous, improved Resolution SWI" (CLEAR-SWI) method.[2]

Additional information on MRI assessment

Macrobleeds and microbleeds were scored on SWI images. cSS was scored on SWI images, the focality score was obtained according to previously published classifications.[3, 4] Perivascular spaces (PVS) were assessed on T2-weighted MRI and Fluid-attenuated inversion recovery (FLAIR) images were used to assess white matter hyperintensities (WMH).

Supplementary references

1. Verberk IMW, Thijssen E, Koelewijn J, et al. Combination of plasma amyloid beta((1-42/1-40)) and glial fibrillary acidic protein strongly associates with cerebral amyloid pathology. Alzheimers Res Ther. 2020 Sep 28;12(1):118.
2. Eckstein K, Bachrata B, Hangel G, et al. Improved susceptibility weighted imaging at ultra-high field using bipolar multi-echo acquisition and optimized image processing: CLEAR-SWI. Neuroimage. 2021 Aug 15;237:118175.
3. Charidimou A, Boulouis G, Roongpiboonsopit D, et al. Cortical superficial siderosis multifocality in cerebral amyloid angiopathy: A prospective study. Neurology. 2017 Nov 21;89(21):2128-35.
4. Charidimou A, Linn J, Vernooij MW, et al. Cortical superficial siderosis: detection and clinical significance in cerebral amyloid angiopathy and related conditions. Brain. 2015 Aug;138(Pt 8):2126-39.
